# Impact of Legislation on Brominated Flame Retardant Concentrations in UK Indoor and Outdoor Environments: Evidence for Declining Indoor Emissions of Some Legacy BFRs

**DOI:** 10.1021/acs.est.3c05286

**Published:** 2024-02-22

**Authors:** Yulong Ma, William A. Stubbings, Jingxi Jin, Reginald Cline-Cole, Mohamed Abou-Elwafa Abdallah, Stuart Harrad

**Affiliations:** † School of Geography, Earth, and Environmental Sciences, University of Birmingham, Birmingham B15 2TT, U.K.; ‡ Department of African Studies & Anthropology, School of History and Cultures, University of Birmingham, Birmingham B15 2TT, U.K.

**Keywords:** house dust, ambient air, deca-BDE, NBFRs, human exposure, HBCDD

## Abstract

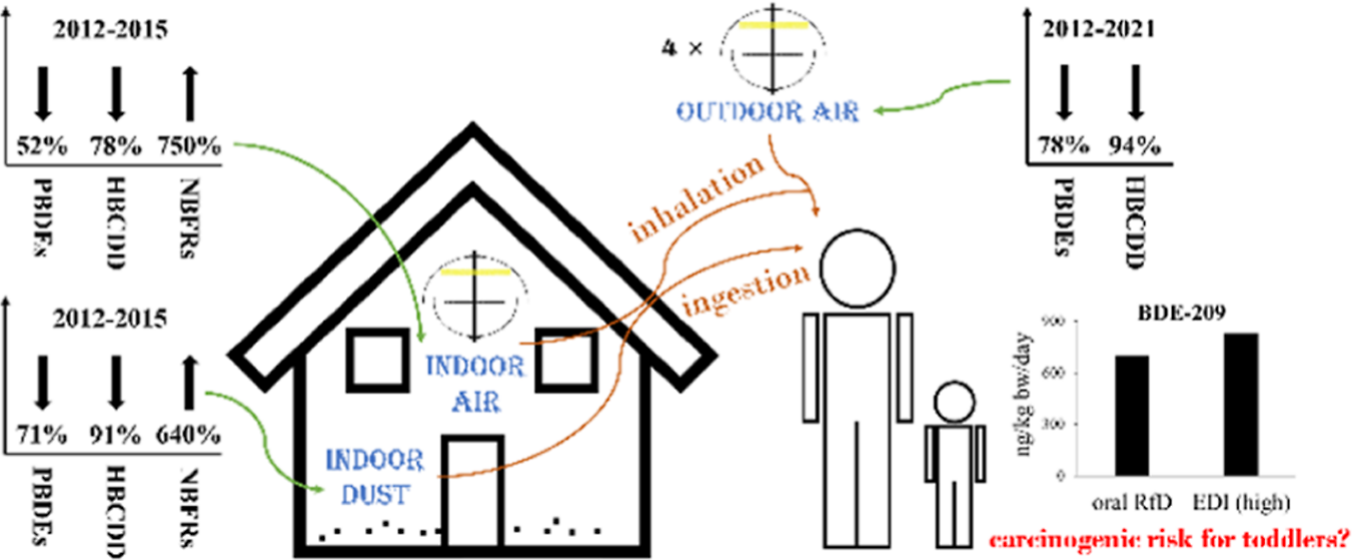

Concentrations of polybrominated diphenyl ethers, hexabromocyclododecane (HBCDD), and novel brominated flame retardants (NBFRs) were measured in indoor dust, indoor air, and outdoor air in Birmingham, UK. Concentrations of ΣBFRs ranged from 490 to 89,000 ng/g, 46–14,000 pg/m^3^, and 22–11,000 pg/m^3^, respectively, in UK indoor dust, indoor air, and outdoor air. BDE-209 and decabromodiphenyl ethane (DBDPE) were the main contributors. The maximum concentration of DBDPE (10,000 pg/m^3^) in outdoor air is the highest reported anywhere to date. In contrast with previous studies of outdoor air in Birmingham, we observed significant correlations between concentrations of tri- to hepta-BDEs and HBCDD and temperature. This may suggest that primary emissions from ongoing use of these BFRs have diminished and that secondary emissions (e.g., evaporation from soil) are now a potentially major source of these BFRs in outdoor air. Conversely, the lack of significant correlations between temperature and concentrations of BDE-209 and DBDPE may indicate that ongoing primary emissions from indoor sources remain important for these BFRs. Further research to clarify the relative importance of primary and secondary sources of BFRs to outdoor air is required. Comparison with earlier studies in Birmingham reveals significant (*p* < 0.05) declines in concentrations of legacy BFRs, but significant increases for NBFRs over the past decade. While there appear minimal health burdens from BFR exposure for UK adults, dust ingestion of BDE-209 may pose a significant risk for UK toddlers.

## Abstract 2

Our observations suggest that further research is required to elucidate whether current and future actions to minimize ongoing indoor sources of BDE-209 and DBDPE may meet with greater success than similar policies aimed at trito-hepta-BDEs and HBCDD.

## Introduction

Polybrominated diphenyl ethers (PBDEs) and hexabromocyclododecane (HBCDD) are two classes of brominated flame retardants (BFRs) which have been ubiquitously used in commercial products. Following the frequent detection of these BFRs in environmental media,^1−10^ biota samples,^11−17^ and even human samples,^12,18−23^ along with reports of adverse effects of these BFRs on the environment and human health,^24−27^ commercial formulations of penta-/octa-BDEs and deca-BDE were banned in Europe in 2004 and 2008, respectively.^28^ Subsequently, penta-/octa-BDEs, HBCDD, and deca-BDE were listed under the Stockholm Convention in 2009, 2014, and 2017, respectively, leading to global phase-out of commercial production and use of these legacy BFRs.^28^ Nevertheless, environmental contamination with legacy BFRs is expected to last for decades due to their persistence, as well as global in-use and waste stocks of PBDEs and HBCDD.^29,30^

This has resulted in increasing demand for novel BFRs (NBFRs) as substitutes for legacy BFRs, with the most commonly used NBFRs being: decabromodiphenyl ethane (DBDPE), bis(2,4,6-tribromophenoxy) ethane (BTBPE), bis(2-ethylhexyl) tetrabromophthalate (BEH-TEBP or TBPH), and 2-ethylhexyl-2,3,4,5-tetrabromobenzoate (EH-TBB or TBB).^28^ To date, NBFRs are less studied in terms of their adverse effects on human health compared to legacy BFRs, yet some previous reports suggested similar or even greater disrupting effects of NBFRs on human hormones compared to PBDEs.^26^

Global restrictions on use of legacy BFRs and increased use of NBFRs should be reflected by temporal changes in BFR concentrations in the environment and biota, a hypothesis suggested in previous studies.^10,12,13,22,31,32^ This has been verified by our recent study reporting increasing NBFR concentrations but decreasing PBDE and HBCDD concentrations in UK foodstuffs.^13^ In terms of the UK indoor environment, a preliminary study compared concentrations of BFRs in UK indoor dust and indoor air in 2015 with earlier UK-based observations and reported that concentrations of some legacy BFRs were decreasing in some microenvironments (e.g., BDE-47 and −99 in office air, BDE-209 in office dust), while concentrations of some NBFRs were increasing (e.g., DBDPE in house dust and office dust).^10^ Unfortunately, further comparisons were not possible,^10^ partly due to differences in the designs of the earlier studies used for comparison (e.g., different sampling strategies), as well as limited historical data on NBFR concentrations available. A more recent study further identified a significant decline in BDE-47 and BDE-99 (but not BDE-209) concentrations that coincided with a significant increase in DBDPE concentrations in UK house dust.^32^ To the best of our knowledge, temporal changes in BFR concentrations in UK outdoor air have only been explored once hitherto.^33^ This earlier study reported slightly higher concentrations of tri- to hepta-BDEs and HBCDD in UK ambient air in 2012 than earlier observations, with no significant differences identified.^33^ Again, differences in study designs limited comparability and further exploration of temporal changes in atmospheric concentrations of legacy BFRs, while temporal changes in NBFR concentrations have, to our knowledge, never been studied in UK ambient air.

To maximize comparability between studies from different years and sampling sites, for indoor dust and indoor air sampling, we adopted the same protocols as used in our 2015 study^10^ and used the outdoor air sampling protocols in an earlier UK-based study.^33^ A total of 8 PBDE congeners (BDE-28, −47, −99, −100, −153, −154, −183, and −209), 9 NBFRs [pentabromobenzene (PBBz), pentabromotoluene (PBT), pentabromoethylbenzene (PBEB), 2,3-dibromopropyl-2,4,6-tribromophenyl ether (DPTE or TBP-DBPE), hexabromobenzene (HBBz), EH-TBB (or TBB), BTBPE, BEH-TEBP (or TBPH), and DBDPE], and 3 HBCDD isomers (α-, β-, and γ-HBCDD) were analyzed in this work. Our aims were to (1) provide an update on BFR concentrations in UK indoor dust, indoor air, and outdoor air; (2) characterize seasonal variations in BFR concentrations in UK ambient air; (3) identify and explain temporal changes in BFR concentrations in UK indoor and outdoor environments in light of recent legislation; and (4) estimate potential health risks posed by BFR exposure to UK citizens.

## Materials and Methods

### Sampling

Floor dust samples (*n* = 30) were collected from living rooms (*n* = 6), kitchens (*n* = 6), and bedrooms (*n* = 18) from 8 UK homes in urban areas in Birmingham during 2021. Dust samples were collected with a portable vacuum cleaner fitted with precleaned nylon mesh filters of 25 μm pore size in the furniture attachment. A 1 m^2^ square area of carpeted floors (or 4 m^2^ for noncarpeted floors) was vacuumed for 2 min (or 4 min in case of noncarpeted floors), before the nylon mesh filters were removed, sealed, and stored at −18 °C. Detailed information on indoor dust sampling has been reported elsewhere.^10^

Indoor air samples (*n* = 30) were collected from the same rooms as indoor dust samples. Passive air samplers (PAS, see Figure S1) were deployed for 28 days in living rooms, kitchens, and bedrooms of 8 UK homes in Birmingham in 2021. Each PAS was equipped with a polyurethane foam disk (PUF, 140 mm diameter, 12 mm thickness, Leicester, UK) and a glass fiber filter (GFF, 12.5 cm diameter, 1 μm pore size, Whatman, UK). Harvested PUFs and GFFs were sealed and stored at 4 °C prior to analysis. Detailed protocols were reported in a previous study.^34^

Outdoor air samples were collected in the backyards of 4 UK homes in Birmingham in 2021 (*n* = 4), and at the Elms Road Observatory Site (EROS, University of Birmingham, Edgbaston, UK) during September 2021 and August 2022 (*n* = 12). Four PAS were deployed at each site for 28 days and then were combined to provide one sample for analysis. Samples at EROS were taken monthly to enable seasonal changes in BFR concentrations to be observed. Harvested PUFs and GFFs were sealed and stored at 4 °C prior to analysis. Detailed information on outdoor air sampling has been published previously.^33^

To clarify, depuration compounds were not used in our PUF–PAS. We used identical passive air sampling rates (Table S1) to those used in the 2015 study for indoor air,^10^ and the 2012 study for outdoor air.^33^ Detailed explanations are given in Supporting Information.

### Analytical Protocols

Protocols for sample preparation and purification have been reported previously.^8−10,33^ Briefly, approximately 200 mg of dust samples was sieved with a precleaned stainless steel test sieve of 500 μm pore size and then spiked with 15 ng of BDE-77, BDE-128, ^13^C-BDE-209, ^13^C-HBBz, ^13^C-EH-TBB, ^13^C-BTBPE, ^13^C-BEH-TEBP, ^13^C-α-HBCDD, ^13^C-β-HBCDD, and ^13^C-γ-HBCDD as internal (surrogate) standards prior to extraction with 2 mL of hexane and acetone (3:1, v/v) for 3 times. The crude extracts were split into two fractions with a florisil column (2 g). Fraction 1 was eluted with 12 mL of hexane and was cleaned with 2 g of acid silica, while fraction 2 was eluted with 15 mL of ethyl acetate and was cleaned with 0.5 g of aminopropyl functionalized silica. The two fractions were then combined and reconstituted into 100 μL of toluene containing 15 ng of ^13^C-BDE-100 and d_18_-γ-HBCDD as recovery determination (syringe) standards prior to GC–MS and LC–MS/MS analysis. Indoor and outdoor air samples (PUFs and GFFs) were also spiked with 15 ng of internal (surrogate) standards and then were extracted on an accelerated solvent extractor (Dionex ASE 350) with hexane and acetone (3:1, v/v). The crude extracts were concentrated to 2 mL prior to purification with 4–6 mL sulfuric acid (95%), after which the extracts were reconstituted into 100 μL of toluene containing 15 ng of recovery determination (syringe) standards prior to instrumental analysis.

Detailed protocols for gas chromatography–mass spectrometry (GC–MS) and high-performance liquid chromatography (HPLC)–MS/MS analysis of BFRs have been reported elsewhere.^13^ Briefly, analysis of PBDEs and NBFRs was conducted on a Trace 1310 GC coupled to an ISQ single quadrupole mass spectrometer (Thermo Scientific, TX, USA) operated with a programmable-temperature vaporizer (PTV) injector. Analysis of HBCDD was conducted on a Shimadzu LC-20AB HPLC (Shimadzu, Kyoto, Japan) equipped with a Varian Pursuit XRS3 C18 (Varian, Inc., Palo Alto, CA, USA) reversed-phase analytical column (150 × 2 mm i.d., 3 μm particle size), coupled to a Sciex API 2000 triple quadrupole mass spectrometer (Applied Biosystems, Foster City, CA, USA).

### QA/QC

Good linearity was obtained from a five-point calibration for all target BFRs (*R*^2^ = 0.9890–0.9999). Limits of quantification (LOQs) for the target BFRs were calculated based on a signal-to-noise ratio of 10 (Table S2). A method blank (200 mg of anhydrous sodium sulfate) was analyzed along with each batch of 6 dust samples. A field blank (a precleaned PUF disk and a preconditioned GFF) was performed at each sampling site when indoor and outdoor PAS were deployed. All target BFRs were detected in the blanks at concentrations below LOQ and thus the samples were not blank corrected. Ten replicate analyses of NIST SRM 2585 (organic contaminants in house dust) were performed prior to sample analyses, revealing average concentrations of BDE-28, −47, −99, −100, −153, −154, −183, and −209 that were 62, 115, 84, 90, 101, 97, 113, and 113% of certified values, respectively. Recoveries of internal standards in all blanks and samples are given in Table S3. Specifically, recoveries of PBDE, NBFR, and HBCDD internal standards were 30–137, 34–113, and 32–115%, respectively, in all blanks and samples.

### Human Exposure Assessment

Inhalation exposure to BFRs of UK citizens was estimated with eq 1
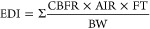
1where EDI is the estimated daily intake of BFRs via inhalation (ng/kg bw/day); BBR is the concentrations of BFRs in indoor or outdoor air (pg/m^3^); AIR is the air inhalation rates; FT is the fraction of time spent in indoor and outdoor environments; and BW is the average body weight of UK citizens.

Estimates of dust ingestion of BFRs for UK people were achieved using eq 2
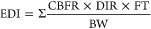
2where EDI is the estimated daily intake of BFRs via dust ingestion (ng/kg bw/day); CBFR is the concentrations of BFRs in house dust (ng/g); DIR is the dust ingestion rates; FT is the fraction of time spent in indoor and outdoor environments; and BW is the average body weight of UK citizens.

### Statistical Analysis

All statistical analyses were conducted with Excel (Microsoft Office 365) and IBM SPSS Statistics 29.0 (Chicago, IL, USA). Because our data did not display normal distribution, the nonparametric Mann–Whitney, Jonckheere-Terpstra, and Wilcoxon signed-rank tests were used to reveal differences between two independent samples, three independent samples, and two related samples, respectively. Only BFRs with a detection frequency (DF) exceeding 50% were included in statistical analyses, with values where BFR concentrations were below LOQ designated as half of LOQ.

## Results and Discussion

### BFR Concentrations in UK Indoor and Outdoor Environments

Concentrations of BFRs in indoor dust, indoor air, and outdoor air collected from Birmingham, UK are summarized in Table 1. All means presented are arithmetic.

**Table 1:** Concentrations of BFRs in Indoor Dust, Indoor Air, and Outdoor Air Collected from Birmingham, UK During 2021 and 2022

	indoor dust (ng/g; *n* = 30)			indoor air (pg/m^3^; *n* = 30)			outdoor air (pg/m^3^; *n* = 16)		
BFRs	DF (%)	median	mean	DF (%)	median	mean	DF (%)	median^a^	mean^a^
BDE-28	20	<0.089	0.20	50	0.33	0.51	81	0.086 (0.085)	0.087 (0.085)
BDE-47	100	3.5	5.8	60	1.0	5.6	94	0.78 (0.76)	0.73 (0.72)
BDE-99	97	3.2	6.3	60	0.98	8.8	88	0.41 (0.39)	0.42 (0.42)
BDE-100	50	0.50	1.1	53	0.46	4.2	100	1.5 (1.5)	1.6 (1.6)
BDE-153	93	1.8	17	3	<0.90	2.3	94	0.22 (0.22)	0.28 (0.25)
BDE-154	93	0.66	9.0	10	<0.56	1.5	81	0.12 (0.12)	0.12 (0.12)
BDE-183	100	4.0	28	33	<0.41	3.5	94	0.14 (0.13)	0.18 (0.15)
BDE-209	100	2300	9900	80	220	460	94	17 (17)	40 (22)
ΣPBDEs		2300	9900		230	480		22 (20)	43 (26)
PBBz	100	1.9	3.3	100	6.5	49	100	0.64 (0.64)	0.61 (0.62)
PBT	100	5.9	24	100	51	150	100	0.80 (0.74)	1.0 (1.1)
PBEB	47	<0.12	0.18	93	0.72	2.0	100	0.44 (0.45)	0.52 (0.52)
DPTE	70	1.1	3.0	20	<0.17	19	81	0.79 (0.79)	0.79 (0.76)
HBBz	100	1.9	2.7	100	3.5	6.0	100	0.33 (0.32)	0.36 (0.34)
EH-TBB	90	4.5	20	70	3.8	10	63	0.14 (0.10)	0.33 (0.34)
BTBPE	83	15	17	33	<3.2	19	69	1.8 (1.5)	9.6 (9.0)
BEH-TEBP	100	190	1000	10	<3.1	40	44	<0.23 (<0.23)	4.8 (4.7)
DBDPE	100	930	2900	70	46	490	94	59 (46)	740 (98)
ΣNBFRs		2200	4000		140	780		81 (63)	760 (120)
ΣHBCDDs^b^		98	730		11	72		1.2 (1.2)	5.6 (5.1)

a Median or mean values in parentheses when the outlier (April 2022 EROS) is excluded.

b Sum of α-, β-, and γ-HBCDD.

#### Indoor Dust

PBDEs were the most abundant BFRs detected in UK house dust, making a mean contribution to BFRs (sum of PBDEs, NBFRs, and HBCDD) of 68%. This was followed by NBFRs, which accounted for 27% on average of total BFRs, while HBCDD only contributed an average of 5%.

PBDE concentrations reported in this study were considerably lower than those reported in Irish homes,^35^ but generally exceeded those reported elsewhere.^5,8,36−43^ Our observations of DBDPE concentrations in UK house dust were lower than those reported in Australian homes^42^ and Irish homes,^35^ but were considerably higher than those reported in other parts of the world.^5,8,37,38,40,43^ NBFR concentrations in house dust from different countries vary greatly, with DBDPE, BEH-TEBP, and EH-TBB frequently detected as the predominant NBFRs.^5,8,36−43^ This is consistent with our observations of NBFR profiles in UK house dust. Concentrations of HBCDD reported in this study were comparable to those reported in house dust collected from China,^41^ but were considerably lower than the concentrations in house dust from Ireland and Spain.^5,35^

Significantly higher concentrations of PBDEs (mean: 14,000 vs 940 ng/g; *p* = 0.002) and HBCDD (mean: 1100 vs 65 ng/g; *p* = 0.002) were observed in floor dust collected from bedrooms than those from kitchens. Likewise, concentrations of NBFRs in floor dust collected from bedrooms were considerably higher than those from kitchens (mean: 4600 ng/g vs 1700 ng/g), although the difference was only marginally significant (*p* = 0.077). These observations were consistent with an earlier UK-based study.^44^ A likely explanation for this could be the carpets frequently used in bedrooms (17 carpeted floors vs 1 noncarpeted floor in this study), i.e., the carpets could either be a source of BFRs or store BFR-containing dust, while carpeted floors were rare in kitchens (all 6 kitchen dust samples were taken from noncarpeted floors in this study). Concentrations of PBDEs (mean: 6200 ng/g), NBFRs (mean: 4300 ng/g), and HBCDD (mean: 130 ng/g) in floor dust collected from living rooms lay between those in bedrooms and kitchens, with no significant differences observed (*p* > 0.05), possibly because carpets were also used in living rooms (2 carpeted floors vs 4 noncarpeted floors in this study), although not as frequently as in bedrooms.

Floor dust samples from two types of residences were collected in this study, i.e., apartments and houses. Concentrations of PBDEs (mean: 17,000 vs 3500 ng/g; *p* = 0.013) and HBCDD (mean: 1400 vs 120 ng/g; *p* = 0.047) in dust collected from houses were significantly higher than those in dust from apartments. Mostly built in the first decade of the 21st century, the apartments examined in this study were constructed more recently than the houses examined, although their specific building years are unclear. These results may reflect the phase-out of legacy BFRs, i.e., PBDEs and HBCDD, in construction materials in the UK in recent decades. In terms of NBFRs, however, concentrations of DBDPE (mean: 3900 ng/g in apartments vs 1700 ng/g in houses) in floor dust from apartments were only marginally significantly higher than those from houses (*p* = 0.093), while no significant differences were observed for total NBFR concentrations (mean: 4200 ng/g in apartments vs 3700 ng/g in houses; *p* = 0.728). This suggested applications of NBFRs as substitutes for legacy BFRs in the construction or refurbishment of both “newer” apartments and “older” houses in the UK.

#### Indoor Air

NBFRs made an average contribution of 59% to total BFR concentrations in indoor air, partially due to more volatile NBFRs (e.g., PBBz and PBT) frequently detected in indoor air. This was followed by PBDEs (mainly BDE-209), contributing 36% on average to total BFRs, while HBCDD only accounted for 5% of total BFRs.

PBDE concentrations in indoor air from UK homes were generally lower than those reported in Ireland^35^ and the US,^43^ but exceeded those reported in Canada,^43^ Spain,^5^ the Czech Republic,^43^ China,^45^ and Japan.^46^ With respect to indoor air concentrations of HBCDD, our observations of UK homes were comparable to earlier observations in Ireland and Japan.^35,46^ Concentrations of DBDPE, the most abundant NBFR, in the indoor air of UK homes were similar to previous observations in Ireland,^35^ China,^45^ and Spain^5^ and were 1 order of magnitude higher than DBDPE concentrations in indoor air collected from the US^43^ and Canada.^43^ Importantly, we note that the highest concentration of DBDPE we observed in UK indoor air (10,000 pg/m^3^) exceeded the maximum DBDPE concentration observed in the indoor air of Irish homes (7000 pg/m^3^)^35^ and is exceeded only by the maximum reported in India of 15,400 pg/m^3^.^47^

No significant differences in BFR concentrations were observed in indoor air from living rooms, kitchens, and bedrooms (*p* > 0.05). This might indicate limited contributions of carpeted floors to BFR concentrations in indoor air, as other factors such as ventilation more likely pose an impact.

Concentrations of PBDEs (mean: 650 vs 340 pg/m^3^) and HBCDD (mean: 100 vs 45 pg/m^3^) observed in the indoor air of “older” houses exceeded considerably those observed in indoor air from “newer” apartments, with such differences statistically significant for PBDEs (*p* = 0.013). By contrast, no significant differences were observed for NBFR concentrations in indoor air from houses and apartments (*p* = 0.918), despite the higher concentrations of total NBFRs in indoor air from apartments than from houses (mean: 1200 pg/m^3^ in apartments vs 310 pg/m^3^ in houses). These observations were consistent with what was observed in indoor dust, and again likely reflect the phase-out of legacy BFRs and increased use of NBFRs as substitutes in the construction industry in the UK.

#### Outdoor Air

The highest BFR concentration, which is also an outlier (i.e., exceeding the average + 2 standard deviations), was observed at EROS at the University of Birmingham in April 2022, i.e., considerably higher concentrations of BDE-209 and DBDPE were identified (BDE-209:300 pg/m^3^ in April 2022 vs 5.1–52 pg/m^3^ in other months; DBDPE: 10,000 pg/m^3^ in April 2022 vs <0.33–370 pg/m^3^ in other months). To our knowledge, this is at least 10 times higher than the highest DBDPE concentrations in outdoor air previously reported elsewhere (e.g., China^3,45,48^ and Spain^2^). We speculate that extensive on-campus construction activities that were conducted to the southwest of EROS may be the source of the elevated concentrations of BDE-209 and DBDPE in outdoor air in this April 2022 sample, as southwest winds prevail across most of the UK. To demonstrate this hypothesis, a back-trajectory analysis of air masses (HYSPLIT, https://www.ready.noaa.gov/HYSPLIT_traj.php, date last accessed: January 06, 2024) was conducted, and the results are given in Figure S2. Further measurement of DBDPE in outdoor air at a wider range of locations would help evaluate whether such elevated concentrations exist elsewhere.

Table 1 compares mean and median concentrations of BFRs in outdoor air before and after the April 2022 sample is excluded. With this sample included, NBFRs (mainly DBDPE) contributed an average of 94% to total BFRs in outdoor air, followed by PBDEs (predominantly BDE-209) and HBCDD (mainly α-HBCDD), which only accounted for 5% and 1%, respectively, of total BFRs in outdoor air. When the April 2022 sample is excluded from consideration, however, the average contributions of NBFRs, PBDEs, and HBCDD to atmospheric concentrations of BFRs were 79%, 18%, and 3%, respectively.

### Correlations between BFR Concentrations in Indoor Dust and Air

Correlations between BFR concentrations in paired indoor dust (*n* = 30) and indoor air (*n* = 30) samples were examined using Spearman’s rho, with test results given in Table S4. Interestingly, while no significant correlations were found for legacy BFRs (i.e., PBDEs and HBCDDs), significant positive correlations were obtained for concentrations of more volatile NBFRs [i.e., those with relatively lower octanol-air partition coefficient (*K*_OA_), including PBBz, PBT, and HBBz] in paired indoor dust and indoor air samples (*p* < 0.01); while for less volatile NBFRs (EH-TBB and DBDPE) such significant correlations were not observed. These findings were consistent with an earlier UK-based study^10^ and were likely to indicate more rapid equilibrium of more volatile NBFRs in the indoor environment.

### Seasonal Variations in BFR Concentrations in Outdoor Air

**Figure 1:**
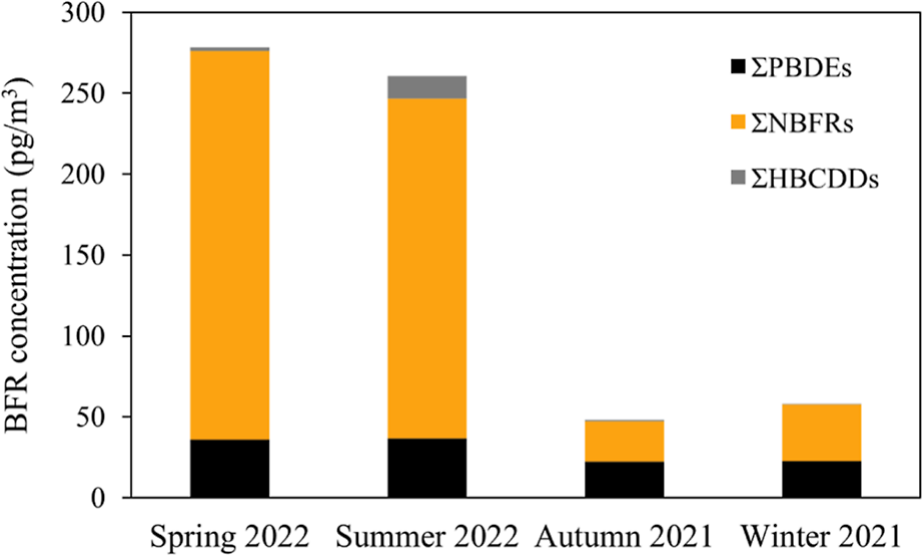
Seasonal variations in atmospheric BFR concentrations observed at EROS (April 2022 excluded). Detailed data can be found in Supporting Information.

Figure 1 depicts the mean atmospheric concentrations of BFRs observed at EROS in different seasons, with detailed data given in Table S5. With or without the April 2022 sample, we observed significantly higher concentrations of most of the target BFRs in spring and summer compared to autumn and winter (*p* < 0.05), including BTBPE, BEH–TEBP, DBDPE, Σ_9_NBFRs (sum of all target NBFRs), Σ_7_PBDEs (sum of BDE-28, −47, −99, −100, −153, −154, and −183), and HBCDD. Atmospheric concentrations of BDE-209 in spring and summer were also considerably higher than those in autumn and winter, although the difference was not statistically significant (*p* > 0.05). Similar findings to these results were reported in South Africa^1^ and China,^48^ possibly suggesting significant impacts of temperature on atmospheric concentrations of BFRs. To confirm this, we calibrated BFR concentrations with an Excel template distributed by the Global Atmospheric Passive Sampling network (Harner, T., 2021 v10 Template for calculating PUF and SIP disk sample air volumes April 28). Significant positive correlations were observed between temperature and calibrated atmospheric concentrations of Σ_7_PBDEs (excluding BDE-209; Spearman’s rho, *r* = 0.588, *p* = 0.044), Σ_8_NBFRs (excluding DBDPE; Spearman’s rho, *r* = 0.680, *p* = 0.015), and HBCDD (Spearman’s rho, *r* = 0.816, *p* = 0.001).

Such temperature-dependence of BFRs other than BDE-209 and DBDPE in outdoor air might be explained by possible impacts of higher ambient temperature on indoor environments, e.g., increased volatilization of BFRs from indoor environments and greater household ventilation at higher temperatures. However, these hypotheses fail to explain the observations of earlier UK studies, where significant seasonal variations in BFR concentrations in outdoor air were not seen.^33,49^ This is likely partly due to indoor heating and cooling systems which minimize seasonal differences in indoor temperature. Some previous studies have postulated that correlations between atmospheric concentrations of BFRs and temperature are an indicator of secondary emissions from, e.g., soil as a major source of atmospheric BFRs.^33,49^ It is plausible, therefore, that the significant correlations between concentrations of tri- to hepta-BDEs and HBCDD and temperature observed here may suggest that primary emissions from ongoing use of these BFRs indoors are becoming less important following bans on their use and that secondary emissions (e.g., evaporation from the soil) are now a potentially major source of tri- to hepta-BDEs and HBCDD in outdoor air. Conversely, the lack of significant correlations between BDE-209 and DBDPE concentrations and temperature may indicate that ongoing primary emissions from consumer products currently constitute a more important source of deca-BDE and DBDPE in the atmosphere compared to secondary emissions. This is consistent with an earlier study conducted in Beijing, China, where low temperature-dependency of particle-phase BDE-209 concentrations in outdoor air was attributed to local ongoing sources rather than volatilization.^50^

We note the relatively small number of samples in this study is a limitation and thus recommend that more detailed studies of the temperature-dependence of BFRs in outdoor air are needed to confirm these findings that have potentially important implications for policy development designed to reduce environmental contamination with BFRs. To illustrate, if the possible interpretation of our data presented above is correct, current and future actions to eliminate/minimize ongoing indoor sources of BDE-209 and DBDPE are likely to meet with greater success than similar policies aimed at trito-hepta-BDEs and HBCDD, for which the majority of emission sources seem no longer indoor.

Other weather conditions apart from temperature were also investigated, including precipitation, air humidity, and wind speed. Interestingly, while no significant correlations were obtained with Spearman’s rho between the number of rainy days or wind speed and atmospheric concentrations of BFRs, significant negative correlations were found between air humidity and outdoor air concentrations of Σ_7_PBDEs (excluding BDE-209; *r* = −0.678, *p* = 0.015), Σ_8_NBFRs (excluding DBDPE; *r* = −0.907, *p* < 0.001), DBDPE (*r* = −0.745, *p* = 0.005) and HBCDD (*r* = −0.900, *p* < 0.001). A negative correlation was also identified between air humidity and atmospheric BDE-209 concentrations, but the correlation was not significant (*r* = −0.316, *p* = 0.316). Atmospheric BFRs tend to accumulate in fine particles,^51^ which are unlikely to be removed by dry or wet deposition.^52^ However, higher air humidity is known to promote the formation and growth of atmospheric aerosols.^53^ This is likely to explain the lack of impact of precipitation, yet the significant impact of air humidity, on BFR concentrations in the atmosphere.

### Temporal Changes in BFR Concentrations in UK Indoor and Outdoor Environments

Temporal changes in mean concentrations of legacy and novel BFRs in UK indoor dust (2015–2021), indoor air (2015–2021), and outdoor air (2012–2021) are shown in Table 2. Detailed data can be found in Table S6.

**Table 2:** Average Percentage of Increase (Positive)/Decrease (Negative) in Mean Concentrations of BFRs in UK Indoor Dust (Mann–Whitney Test), Indoor Air (Mann–Whitney Test), and Outdoor Air (Wilcoxon Signed-Rank Test)^c^

BFRs	indoor dust (2015–2021)	indoor air (2015–2021)	outdoor air (2012–2021)
BDE-28	–89%	–98%^b^	–98%^a^
BDE-47	–59%^b^	–95%^b^	–89%^a^
BDE-99	–80%^b^	–93%^b^	–93%
BDE-100	–74%^b^	–91%	–68%
BDE-153	+250%	–91%	–97%^a^
BDE-154	+350%	–90%	–99%^a^
BDE-183	+280%	+26%	–75%^a^
BDE-209	–71%	–31%	–75%
ΣPBDEs	–71%	–52%	–78%^a^
PBBz	0%	+640%^a^	n.a.
PBT	+240%^b^	+760%^a^	n.a.
PBEB	–92%^b^	+27%^b^	n.a.
DPTE	–55%	+450%^b^	n.a.
HBBz	+51%^b^	–45%	n.a.
EH-TBB	–5%	+110%	n.a.
BTBPE	+21%	+69%	n.a.
BEH-TEBP	+320%^b^	+300%^a^	n.a.
DBDPE	+1100%^b^	+1800%^a^	n.a.
ΣNBFRs	+640%^b^	+750%^a^	n.a.
α-HBCDD	–89%^b^	–13%	–92%^a^
β-HBCDD	–93%^b^	–8%	–95%^a^
γ-HBCDD	–92%^b^	–93%^b^	–95%^a^
ΣHBCDDs	–91%^b^	–78%^b^	–94%^a^

a Significant difference at 0.05 level (*p* < 0.05).

b Significant difference at 0.01 level (*p* < 0.01).

c n.a. not available due to limited data on NBFR concentrations in 2012.

#### Legacy BFRs

Considerable declines in concentrations of most of the target legacy BFRs (PBDEs and HBCDD) were observed in UK indoor and outdoor environments. Mean concentrations of PBDEs (predominantly BDE-209) dropped by 71, 52, and 78% in UK indoor dust (2015–2021), indoor air (2015–2021), and outdoor air (2012–2021), respectively, and such changes in PBDE concentrations in UK ambient air were statistically significant (*p* < 0.05). With respect to individual PBDE congeners, significant decreases were observed for BDE-47, BDE-99, and BDE-100 in indoor dust (*p* < 0.01), for BDE-28, BDE-47, and BDE-99 in indoor air (*p* < 0.01), and for BDE-28, BDE-47, BDE-153, BDE-154, and BDE-183 in outdoor air (*p* < 0.05). Mean concentrations of BDE-209 also decreased considerably in UK indoor dust (by 71%), indoor air (by 31%), and outdoor air (by 75%), but the differences were not significant (*p* > 0.05). These results likely reflect the much later global phase-out of deca-BDE in commercial products compared to penta- and octa-BDEs. Not surprisingly, concentrations of HBCDD also declined significantly in UK indoor dust (*p* < 0.01) and indoor air (*p* < 0.01) during 2015 and 2021, and in UK outdoor air (*p* < 0.05) during 2012 and 2021. It is notable that earlier studies conducted in the UK failed to observe any significant changes in HBCDD concentrations in indoor and outdoor samples,^10,32^ suggesting that decreases in indoor contamination of HBCDD were slow and that insufficient time had elapsed for significantly declined HBCDD concentrations to be observed when the studies were conducted.^32^ Therefore, this is the first report of significantly declining indoor and outdoor concentrations of HBCDD in the UK, suggesting active impacts on UK indoor and outdoor environments of restrictions on HBCDD use.

#### NBFRs

We observed a significant increase in NBFR concentrations in UK indoor dust (*p* < 0.01) and indoor air (*p* < 0.05) between 2015 and 2021. Mean concentrations of total NBFRs increased by 640 and 750%, respectively, in UK indoor dust and indoor air. The greatest increase was observed for DBDPE, the predominant NBFR. Mean concentrations of DBDPE in UK house dust increased from 240 o 1500 ng/g during 2015 and 2019,^10,32^ reaching 2900 ng/g in 2021, i.e., an increase by 1100% between 2015 and 2021 (*p* < 0.01). DBDPE concentrations also increased by 1800% in UK indoor air (*p* < 0.05) during 2015 and 2021. This could be attributed to the extensive use of DBDPE as a replacement for deca-BDE in recent years.^28^ Mean concentrations of BTBPE and BEH-TEBP, substitutes for penta- and octa-BDEs, also increased by 21 and 320% in indoor dust and by 69 and 300% in indoor air, respectively; these changes were significant for BEH-TEBP (indoor dust: *p* < 0.01; indoor air: *p* < 0.05). Significant increases were also found in PBBz concentrations in indoor air (*p* < 0.05), in PBT concentrations in indoor dust (*p* < 0.01) and indoor air (*p* < 0.05), in PBEB and DPTE concentrations in indoor air (*p* < 0.01), and in HBBz concentrations in indoor dust (*p* < 0.01). While this might be due to their increasing consumption in the UK (data on UK consumption of these NBFRs is scarce), these bromobenzenes were more likely to be degradation products of deca-BDE or DBDPE.^54,55^ With respect to outdoor air, no reports other than the 2012 study^33^ were identified of atmospheric concentrations of the target NBFRs in the UK. Most of the target NBFRs were not detected in UK ambient air in that study, with BEH–TEBP and DBDPE the only exceptions detected in 33 and 50% of the UK outdoor air samples, respectively.^33^ Therefore, concentrations of the target NBFRs were not reported in this earlier study, making it hard to evaluate temporal changes in atmospheric NBFR concentrations in the UK. Notwithstanding this, the much higher detection frequencies of NBFRs in UK ambient air observed in the present study compared to those in the 2012 study potentially suggest concentrations of NBFRs in UK outdoor air have risen over the past decade. Further investigations are encouraged to confirm this finding.

Notwithstanding the limited number of samples analyzed, this work presents potentially important findings that global phase-out of legacy BFRs has been reflected in considerably reduced concentrations of legacy BFRs and significantly increased concentrations of NBFRs in UK indoor and outdoor environments.

### Estimates of Human Exposure to BFRs for UK Residents

Mean dust ingestion rates of 20 mg/day and 50 mg/day were assumed for UK adults (≥16 years old) and toddlers (≤3 years old), respectively,^56−58^ under the mean (median) exposure scenario where mean (median) concentrations of BFRs in house dust were applied; while high dust ingestion rates of 50 and 200 mg/day were assumed for UK adults and toddlers, respectively,^56−58^ under the high exposure scenario alongside application of 95th percentile BFR concentrations in house dust. With respect to inhalation exposure to BFRs, mean, median, and 95th percentile concentrations of BFRs in indoor (outdoor) air were adopted to generate the mean, median, and high estimates of exposure to BFRs via indoor (outdoor) air inhalation. Air inhalation rates of 20 and 3.8 m^3^/day were assumed for UK adults and toddlers, respectively.^10^

#### Dust Ingestion

Estimates of dust ingestion of BFRs for UK adults and toddlers are summarized in Tables S7 and S8. Mean (median) exposure to total BFRs via dust ingestion was estimated to be 2.8 ng/kg bw/day (1.3 ng/kg bw/day) and 61 ng/kg bw/day (30 ng/kg bw/day) for UK adults and toddlers, respectively, with the high exposure estimates of 30 and 1000 ng/kg bw/day. PBDEs (mainly BDE-209) dominated dust ingestion of BFRs, accounting for 68% on average of dust ingestion of total BFRs. This was followed by NBFRs (primarily DBDPE and BEH–TEBP) and HBCDD, which contributed an average of 27 and 5%, respectively, to dust ingestion of BFRs.

#### Inhalation

Human inhalation exposure to BFRs was estimated to be 0.25 ng/kg bw/day (0.11 ng/kg bw/day) and 0.41 ng/kg bw/day (0.19 ng/kg bw/day), respectively, for UK adults and toddlers under the mean (median) exposure scenario. The high exposure scenario estimates of BFRs were 0.60 and 0.98 ng/kg bw/day for UK adults and toddlers, respectively. Inhalation of indoor air contributed the major proportion (93% mean) of inhalation exposure to BFRs, partly due to the higher concentrations of BFRs observed in indoor air compared to outdoor air, accentuated by the longer proportion of time spent indoors. Unlike dust ingestion of BFRs, NBFRs contributed most to inhalation exposure to BFRs (61% mean), followed by PBDEs (34% mean) and HBCDD (5% mean).

#### Comparisons with Dietary Intake

**Figure 2:**
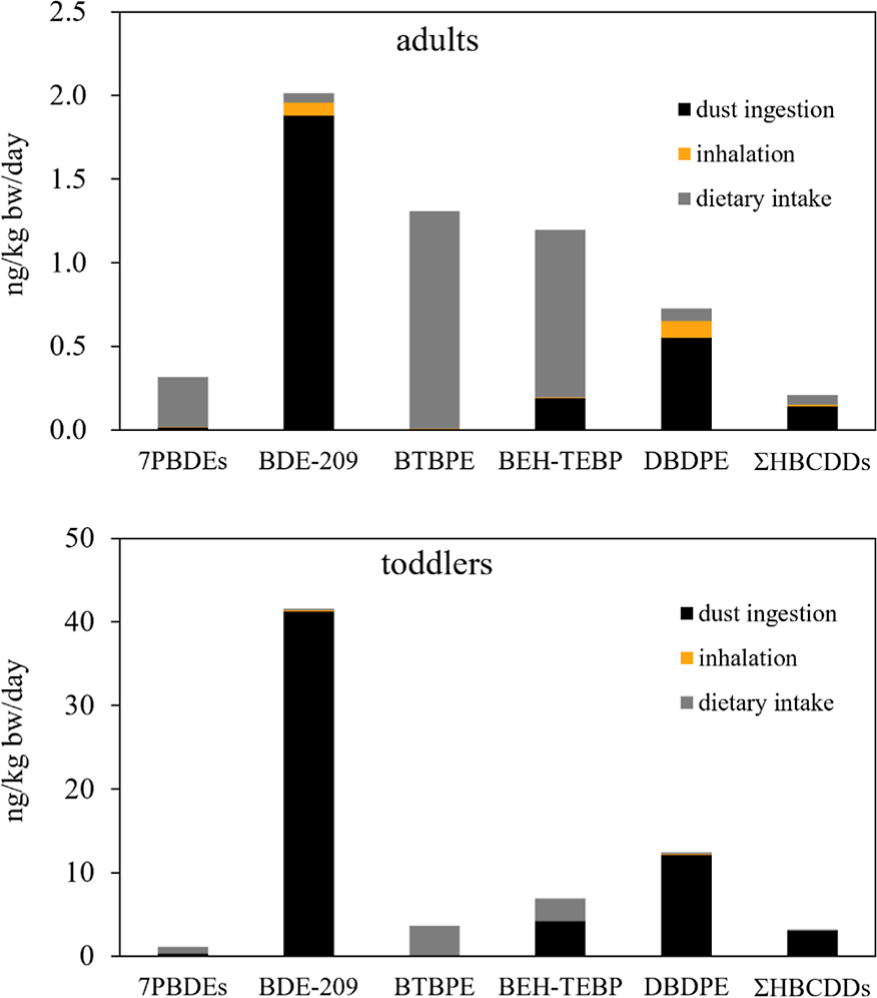
Estimated mean daily intake of BFRs via dust ingestion (this study), inhalation (this study), and dietary intake^13^ for UK adults and toddlers. 7PBDEs are the sum of BDE-28, −47, −99, −100, −153, −154, and −183. Detailed data can be found in Supporting Information.

We have previously investigated human dietary intake of BFRs for UK residents,^13^ summarized in Tables S7 and S8. Under the mean exposure scenario, human exposure to BFRs (sum of dust ingestion, inhalation, and dietary intake) was estimated to be 5.9 and 69 ng/kg bw/day for adults and toddlers, with high estimates of 48 and 1100 ng/kg bw/day, respectively. Figure 2 compares the most abundant BFRs when the three exposure pathways were combined. Based on the mean estimates, dust ingestion contributed most to human exposure to BDE-209 (93% for adults and 99% for toddlers), DBDPE (76% for adults and 97% for toddlers), and HBCDD (67% for adults and 96% for toddlers) compared to inhalation exposure and dietary intake. By contrast, dietary intake accounted for 9% and 99% of adults’ exposure to tri- to hepta-BDEs and BTBPE and accounted for 7% and 98% of toddlers’ exposure to tri- to hepta-BDEs and BTBPE, respectively. This is likely due to the higher dust concentrations and lower bioavailability^13^ of BDE-209, DBDPE, and HBCDD compared to tri- to hepta-BDEs and BTBPE.

#### Comparisons with Earlier Studies in the UK

Table 3 compares mean BFR exposure doses estimated for UK residents between 2015 and 2021. Total exposure (sum of dust ingestion, inhalation exposure, and dietary intake) to PBDEs and HBCDD estimated for UK toddlers declined from 100 to 42 ng/kg bw/day and from 35 to 3.2 ng/kg bw/day during 2015 and 2021, respectively. For adults, the estimated exposure doses also decreased from 7.4 to 2.3 ng/kg bw/day for PBDEs and from 2.3 to 0.21 ng/kg bw/day for HBCDD, respectively. This highlights the positive impact of regulatory efforts aimed at controlling legacy BFRs in the UK. In the meantime, human exposure to NBFRs increased from 6.3 to 24 ng/kg bw/day for UK toddlers, and from 2.0 to 3.4 ng/kg bw/day for UK adults, respectively, possibly reflecting greater health burdens posed by the use of NBFRs as substitutes in the UK.

**Table 3:** Temporal Changes in Estimated Mean Exposure to BFRs for UK Residents between 2015 and 2021 (ng/kg bw/day)

	mean exposure estimates in 2015^a^				mean exposure estimates in 2021			
BFRs	dust ingestion^10^	inhalation^10^	dietary intake^12^	total exposure	dust ingestion	inhalation	dietary intake^13^	total exposure
Toddlers								
ΣPBDEs	100	0.17	4.2	100	41	0.14	1.0	42
ΣNBFRs	3.7	0.029	2.6	6.3	17	0.25	6.8	24
ΣHBCDDs	34	0.027	0.88	35	3.0	0.021	0.12	3.2
Total BFRs	140	0.22	7.7	150	61	0.41	7.9	69
Adults								
ΣPBDEs	5.5	0.12	1.8	7.4	1.9	0.083	0.36	2.3
ΣNBFRs	0.72	0.026	1.3	2.0	0.75	0.15	2.5	3.4
ΣHBCDDs	1.8	0.076	0.44	2.3	0.14	0.012	0.057	0.21
Total BFRs	8.0	0.22	3.5	12	2.8	0.25	2.9	5.9

a Data converted from ng/day to ng/kg bw/day.

#### Health Risk Assessment

The EDIs of most of the target BFRs both for UK adults and for toddlers were well below the corresponding reference doses (RfDs), reflected by EDI/RfD ratios that are much smaller than 1 for most BFRs (Table S9). This indicates minimal health burdens posed by exposure to BFRs for UK populations. However, it is noticeable that the US EPA has assigned an oral slope factor for carcinogenic risk of 700 (ng/kg bw/day)^−1^ for BDE-209.^59^ This was exceeded by the EDI of BDE-209 for UK toddlers (830 ng/kg bw/day) under the high exposure scenario. Importantly, we do not include all possible pathways of BFR exposure in this study (e.g., dermal uptake of BFRs was not investigated). The importance of dermal uptake of BFRs via contact with furniture fabrics, particularly in summer, has been highlighted previously.^60^ For instance, dermal uptake of penta-BDEs via contact with furniture fabrics was estimated to exceed overall exposure to penta-BDEs via other pathways for American adults during summer, while for HBCDD the estimated dermal uptake of UK adults and toddlers exceeded the reported mean daily intakes via other pathways for these UK age groups.^60^ This suggests that total exposure to BFRs could be much higher than our estimates for UK adults and toddlers, raising deeper concerns over potential health risks posed by BFRs to toddlers, with BDE-209 of particular interest. Further studies are needed to evaluate this more fully.

## Supporting Information Available

Additional experimental details, materials, methods, and statistics, including QA/QC protocols and human exposure estimates (PDF)
