# Supplementary material for: Impact of Legislation on Brominated Flame Retardant Concentrations in UK Indoor and Outdoor Environments: Evidence for Declining Indoor Emissions of Some Legacy BFRs
Source: Environ Sci Technol. 2024 Feb 22;58(9):4237–46. doi: 10.1021/acs.est.3c05286 (PMC10919073; doi:10.1021/acs.est.3c05286)
Supplement: Supplementary file 1 — es3c05286_si_001.pdf [file es3c05286_si_001.pdf]

Impact of legislation on brominated flame retardant (BFR) concentrations in UK indoor and outdoor environments: evidence for declining indoor emissions of some legacy BFRs

Supporting Information

(17 pages, 9 tables, and 3 figures)

Yulong Ma [a] *, William A. Stubbings [a], Jingxi Jin [a], Reginald Cline-Cole [b],

Mohamed Abou-Elwafa Abdallah [a], Stuart Harrad [a]

[a] School of Geography, Earth, and Environmental Sciences,

University of Birmingham,

Birmingham B15 2TT,

UK

[b] Department of African Studies & Anthropology, School of History and Cultures,

University of Birmingham,

Birmingham, B15 2TT,

UK

* Corresponding author. E-mail address: mayl1316@163.com (Y. Ma)

Passive air sampling rates

Indoor air sampling rates. Indoor air sampling rates for PBDEs and HBCDDs were obtained from a previous study,[1] as the same PAS units to this earlier study were used in our study. Specifically, the following equation was used for calculating sampling rates of PBDEs and HBCDD:[1]

$$SR = Sgas \times Pgas + Sparticle \times Pparticle \qquad (S1)$$

where SR is sampling rates of BFRs (m^3/day); $Sgas$ and $Sparticle$ are sampling rates of gaseous and particle BFRs, respectively, and the data were obtained from Abdallah's study (m^3/day);[2] $Pgas$ and $Pparticle$ are percentages of target BFRs in gaseous phase and particle phase, respectively, and the data were obtained from Abdallah's study.[2]

The sampling rates for NBFRs were assumed equivalent to literature reported sampling rates for PBDEs of similar K_{OA} and K_{OW} values.[1, 3] This makes our observations more comparable to previous observations so that temporal changes in BFR concentrations in indoor air could be identified. Indoor air sampling rates for all the target BFRs are summarized in Table S1.

Outdoor air sampling rates. Outdoor air sampling rates for PBDEs have been calculated in an earlier study.[3] Specifically, PAS units were deployed for 50 days and were collected at 10 day intervals, while active air samplers were also deployed for calibration purpose. Total volume of air sampled by PAS was calculated by the equation below:[3]

$$V = \frac{M}{C} \qquad (S2)$$

where V is the total volume of air sampled by PAS (m^3); M is the mass of a particular PBDE congener detected in PAS (pg); C is the concentration of a particular PBDE congener in outdoor air (pg/m^3), detected by active air samplers.

To calculate outdoor air sampling rates for PBDEs, V was plotted against sampling period (days) and the sampling rates were then calculated from the slope of the line.[3] Following this method, outdoor air sampling rates for PBDEs were estimated to be 2.26 m^3/day for deca-BDE and 3.92 m^3/day for tri- to hepta- BDEs.[3]

We adopted the same sampling rates for PBDEs in our work because the same PAS units and the same sampling site to that earlier study were used in our study. Further, following the same rules, the sampling rates for NBFRs were assumed equivalent to literature reported sampling rates for PBDEs of similar K_{OA} and K_{OW} values.[1, 3] (Table S1). This is to ensure that our observations are comparable to previous observations so that temporal changes in BFR concentrations in outdoor air could be identified.

Adjustment of outdoor air sampling rates. An Excel template distributed by the Global Atmospheric Passive Sampling (GAPS) network was used to adjust air sampling rates of outdoor PAS in this study.[4] This enables impacts of ambient air temperature on air sampling rates of PAS to be evaluated, as higher temperature is likely to result in increased molecular diffusion and greater shifts to gas-particle partitioning in ambient air. Fig. S3 compares concentrations of tri-to-hepta-PBDEs and NBFRs before and after air sampling rates of PAS were adjusted with temperature (BDE-209 and HBCDD are not included in the template, and thus are not compared). The adjusted concentrations of PBDEs and NBFRs were very consistent with the pre-adjusted concentrations, with the variations below 1%. Therefore, outdoor air sampling rates were not adjusted in this study when comparing our observations with previous studies. This is to ensure that our observations are more comparable to previous reports.

Estimated human exposure to BFRs via inhalation and dust ingestion.

Typical activity patterns were assumed for UK population, i.e., 10% of time spent outdoors and 90% of time spent indoors for both adults (≥ 16 years old) and toddlers (≤ 3 years old).[5] For adults a working time of 8 h per day was assumed during weekdays, while the rest of the time spent indoors was assumed to be at home. For toddlers all the time spent indoors was assumed to be at home. Therefore, 10% of time spent outdoors, 66% of time spent at home, and 24% of time spent in office (which was not included in this study) were assumed for UK adults in terms of inhalation exposure to BFRs; and 10% of time spent outdoors and 90% of time spent at home were assumed for UK

toddlers in terms of inhalation exposure to BFRs. With respects to indoor dust ingestion of BFRs, dust ingestion was assumed to occur pro-rata to typical indoor activity patterns, i.e., 74% home and 26% office for adults, and 100% home for toddlers. An absorption rate of 100% was assumed for UK adults and toddlers when estimating human exposure to BFRs via inhalation and dust ingestion.[1] The average body weight of UK adults and toddlers was assumed to be 78 kg and 12 kg, respectively.[6]

Tables

Table S1. Passive air sampling rates for each analyte

BFRs	Indoor air sampling rates (m^3/day)[1]	Outdoor air sampling rates (m^3/day)[3]
BDE-28	1.74	3.92
BDE-47	1.472	3.92
BDE-99	0.992	3.92
BDE-100	1.014	3.92
BDE-153	0.685	3.92
BDE-154	0.691	3.92
BDE-183	0.550	3.92
BDE-209	0.567	2.26
PBBz	1.74	3.92
PBT	1.74	3.92
PBEB	1.472	3.92
DPTE	1.74	3.92
HBBz	1.472	3.92
EH-TBB	0.688	3.92
BTBPE	0.550	3.92
BEH-TEBP	0.567	2.26
DBDPE	0.567	2.26
α-HBCDD	0.807	3.92
β-HBCDD	0.775	3.92
γ-HBCDD	0.761	3.92

Table S2. Limits of quantification (LOQs) for each analyte for indoor dust, indoor air, and outdoor air analysis

BFRs	Method LOQs for indoor dust (ng/g)	Method LOQs for indoor air (pg/m^3)	Method LOQs for outdoor air (pg/m^3)
BDE-28	0.22	0.32	0.036
BDE-47	0.15	0.13	0.012
BDE-99	0.19	0.41	0.026
BDE-100	0.18	0.46	0.030
BDE-153	0.14	0.90	0.039
BDE-154	0.11	0.56	0.025
BDE-183	0.26	0.41	0.014
BDE-209	1.7	3.2	0.20
PBBz	0.11	0.11	0.013
PBT	0.22	0.14	0.016
PBEB	0.15	0.15	0.014
DPTE	0.15	0.17	0.019
HBBz	0.15	0.22	0.020
EH-TBB	0.19	0.96	0.042
BTBPE	0.74	3.3	0.11
BEH-TEBP	1.1	3.2	0.20
DBDPE	1.8	3.9	0.26
α-HBCDD	0.54	2.2	0.11
β-HBCDD	0.51	2.2	0.11
γ-HBCDD	0.36	1.6	0.076

Table S3. Recoveries of internal standards (I.S.) in method blanks (n = 5), field blanks (n = 8), dust samples (n = 30), indoor air samples (n = 30), outdoor air samples (n = 16), and NIST SRM 2585 dust (n = 10)

I.S.	Method blanks	Field blanks	Dust samples	Indoor air samples	Outdoor air samples	SRMs
BDE-77	72-75%	63-78%	67-94%	50-84%	55-80%	67-86%
BDE-128	45-53%	46-64%	40-101%	35-74%	43-67%	62-81%
^{13}C-BDE-209	98-113%	30-55%	52-137%	30-48%	32-53%	34-118%
^{13}C-HBBz	63-69%	57-69%	69-113%	35-85%	35-76%	-
^{13}C-EH-TBB	48-55%	79-95%	49-79%	70-99%	66-104%	-
^{13}C-BTBPE	40-47%	39-57%	42-88%	34-60%	39-55%	-
^{13}C-BEH-TEBP	49-55%	37-61%	42-64%	35-60%	39-72%	-
^{13}C-α-HBCDD	64-86%	54-99%	32-68%	32-98%	34-93%	-
^{13}C-β-HBCDD	67-81%	64-115%	36-103%	50-107%	34-73%	-
^{13}C-γ-HBCDD	80-84%	87-111%	66-111%	53-113%	72-93	-

Table S4. Correlation coefficients (Spearman's rho) of BFR concentrations between indoor and outdoor environments

BFRs	lg K_{OA}[1]	Indoor air vs indoor dust [a]
BDE-28	9.50	n.a.
BDE-47	10.53	0.175
BDE-99	11.31	0.317
BDE-100	11.13	0.190
BDE-153	11.82	n.a.
BDE-154	11.92	n.a.
BDE-183	11.96	n.a.
BDE-209	15.27	0.185
PBBz	9.10	0.764**
PBT	9.66	0.856**
PBEB	9.97	n.a.
DPTE	8.01	n.a.
HBBz	10.26	0.776**
EH-TBB	12.34	0.075
BTBPE	15.67	n.a.
BEH-TEBP	16.86	n.a.
DBDPE	19.22	0.153
α-HBCDD	14.43	0.014
β-HBCDD	14.64	0.271
γ-HBCDD	14.17	n.a.

[a] only BFRs with a DF exceeding 50% were included.

* $p < 0.05$.

** $p < 0.01$.

Table S5. Arithmetic mean atmospheric concentrations of BFRs observed at EROS in different seasons (pg/m^3)

BFRs	Autumn 2021	Winter 2021	Spring 2022 [a]	Summer 2022
Σ_7PBDEs	3.3	2.8	4.2 (4.1)	4.7
BDE-209	19	20	120 (32)	32
PBBz	0.78	0.39	0.60 (0.65)	0.94
PBT	0.90	0.55	0.82 (0.80)	2.1
PBEB	0.73	0.51	0.43 (0.44)	0.77
DPTE	1.0	0.78	0.98 (0.79)	1.1
HBBz	0.60	0.31	0.42 (0.33)	0.36
EH-TBB	0.12	0.11	0.15 (0.14)	0.091
BTBPE	<0.11	5.2	12 (8.6)	26
BEH-TEBP	3.1	<0.20	7.8 (9.0)	14
DBDPE	18	27	3,600 (220)	160
Σ_9NBFRs	25	35	3,600 (240)	210
Σ_3HBCDDs	0.89	0.48	5.6 (2.3)	14

[a] arithmetic mean concentrations in parentheses when the April 2022 sample is excluded.

Table S6. Temporal changes in arithmetic mean concentrations of BFRs in UK indoor and outdoor environments

BFRs	Indoor dust (ng/g)		Indoor air (pg/m^3)		Outdoor air (pg/m^3) [a]	
	**2015[1]**	**2021**	**2015[1]**	**2021**	**2012[3]**	**2021**
BDE-28	1.9	0.20	22	0.51	5.4	0.093
BDE-47	14	5.8	120	5.6	7.6	0.83
BDE-99	31	6.3	130	8.8	7.6	0.54
BDE-100	4.2	1.1	44	4.2	5.4	1.7
BDE-153	4.8	17	24	2.3	11	0.28
BDE-154	2.0	9.0	14	1.5	13	0.14
BDE-183	7.4	28	2.8	3.5	0.75	0.19
BDE-209	34,000	9,900	660	460	110	29
**Σ_8PBDEs**	**34,000**	**9,900**	**1,000**	**480**	**150**	**33**
PBBz	3.3	3.3	6.6	49		0.72
PBT	7.1	24	17	150		1.1
PBEB	2.3	0.18	1.6	2.0		0.65
DPTE	6.6	3.0	3.5	19		1.0
HBBz	1.8	2.7	11	6.0		0.43
EH-TBB	21	20	4.8	10		0.075
BTBPE	14	17	11	19		11
BEH-TEBP	240	1,000	10	40		5.8
DBDPE	240	2,900	26	490		59
**Σ_9NBFRs**	**540**	**4,000**	**92**	**780**		**80**
α-HBCDD	2,300	250	43	37	42	3.4
β-HBCDD	1,100	73	17	16	22	1.1
γ-HBCDD	4,900	400	270	19	22	1.1
**Σ_3HBCDDs**	**8,300**	**730**	**330**	**72**	**86**	**5.6**

[a] data obtained in certain months (June 2012 to January 2013 vs June 2021 to January 2022) at EROS are compared.

Table S7. Estimated daily intake of BFRs via inhalation, dust ingestion, and dietary intake for UK adults (ng/kg bw/day)

BFRs	Indoor dust ingestion			Inhalation of indoor air			Inhalation of outdoor air			Dietary intake[7]		Total exposure	
	**Mean**	**Median**	**High**	**Mean**	**Median**	**High**	**Mean**	**Median**	**High**	**Mean**	**High**	**Mean**	**High**
Σ_7PBDEs [a]	0.012	0.0025	0.079	0.0045	0.0013	0.0038	0.000088	0.000082	0.00014	0.30	2.1	0.32	2.2
BDE-209	1.9	0.44	24	0.077	0.037	0.31	0.0010	0.00044	0.0029	0.057	0.39	2.0	24
**Σ_8PBDEs**	**1.9**	**0.44**	**24**	**0.082**	**0.040**	**0.34**	**0.0011**	**0.00056**	**0.0030**	**0.36**	**2.5**	**2.3**	**26**
PBBz	0.00063	0.00035	0.0061	0.0083	0.0011	0.042	0.000016	0.000016	0.000026	0	0	0.0089	0.048
PBT	0.0046	0.0011	0.055	0.025	0.0086	0.10	0.000027	0.000021	0.000060	0	0	0.029	0.15
PBEB	0.000034	0.000023	0.00034	0.00034	0.00012	0.0017	0.000013	0.000011	0.000021	0	0	0.00039	0.0020
DPTE	0.00057	0.00021	0.0056	0.0032	0.000029	0.0036	0.000020	0.000020	0.000035	0.075	0.45	0.079	0.46
HBBz	0.00052	0.00035	0.0031	0.0010	0.00059	0.0036	0.0000092	0.0000086	0.000017	0	0	0.0015	0.0067
EH-TBB	0.0038	0.00085	0.057	0.0017	0.00065	0.0084	0.0000085	0.0000036	0.000040	0.059	0.35	0.064	0.42
BTBPE	0.0032	0.0028	0.017	0.0031	0.00054	0.017	0.00025	0.000046	0.00090	1.3	8.1	1.3	8.1
BEH-TEBP	0.19	0.036	3.1	0.0067	0.00052	0.055	0.00012	0.0000059	0.00036	1.0	6.0	1.2	9.2
DBDPE	0.55	0.18	4.2	0.083	0.0077	0.18	0.019	0.0015	0.073	0.073	0.44	0.72	4.8
**Σ_9NBFRs**	**0.75**	**0.43**	**5.2**	**0.13**	**0.024**	**0.30**	**0.019**	**0.0021**	**0.074**	**2.5**	**15**	**3.4**	**21**
**HBCDD**	**0.14**	**0.019**	**2.1**	**0.012**	**0.0018**	**0.058**	**0.00014**	**0.000032**	**0.00065**	**0.057**	**0.48**	**0.21**	**2.6**
**Σ_{20}BFRs**	**2.8**	**1.3**	**30**	**0.23**	**0.11**	**0.52**	**0.021**	**0.0026**	**0.077**	**2.9**	**18**	**5.9**	**48**

[a] sum of BDE-28, -47, -99, -100, -153, -154, and -183.

Table S8. Estimated daily intake of BFRs via inhalation, dust ingestion, and dietary intake for UK toddlers (ng/kg bw/day)

BFRs	Indoor dust ingestion			Inhalation of indoor air			Inhalation of outdoor air			Dietary intake[7]		Total exposure	
	Mean	Median	High	Mean	Median	High	Mean	Median	High	Mean	High	Mean	High
Σ_7PBDEs [a]	0.27	0.056	2.8	0.0075	0.0021	0.0064	0.00011	0.00010	0.00018	0.83	5.9	1.1	8.7
BDE-209	41	9.6	830	0.13	0.062	0.53	0.0013	0.00055	0.0036	0.16	1.1	41	830
**Σ_8PBDEs**	**41**	**9.6**	**830**	**0.14**	**0.067**	**0.57**	**0.0014**	**0.00069**	**0.0038**	**1.0**	**7.0**	**42**	**840**
PBBz	0.014	0.0077	0.21	0.014	0.0019	0.070	0.000019	0.000020	0.000032	0	0	0.028	0.28
PBT	0.10	0.025	1.9	0.041	0.015	0.17	0.000033	0.000025	0.000074	0	0	0.14	2.1
PBEB	0.00075	0.00050	0.012	0.00058	0.00021	0.0028	0.000016	0.000014	0.000026	0	0	0.0014	0.015
DPTE	0.013	0.0046	0.20	0.0055	0.000048	0.0060	0.000025	0.000025	0.000043	0.20	1.2	0.22	1.4
HBBz	0.011	0.0077	0.11	0.0017	0.0010	0.0060	0.000011	0.000011	0.000022	0	0	0.013	0.11
EH-TBB	0.083	0.019	2.0	0.0028	0.0011	0.014	0.000011	0.0000044	0.000049	0.16	0.96	0.25	3.0
BTBPE	0.071	0.063	0.59	0.0053	0.00091	0.029	0.00030	0.000057	0.0011	3.6	22	3.7	23
BEH-TEBP	4.2	0.79	110	0.011	0.00088	0.093	0.00015	0.0000073	0.00045	2.7	16	6.9	130
DBDPE	12	3.9	150	0.14	0.013	0.30	0.023	0.0019	0.091	0.19	1.2	12	150
**Σ_9NBFRs**	**17**	**9.4**	**180**	**0.22**	**0.040**	**0.51**	**0.024**	**0.0026**	**0.091**	**6.8**	**42**	**24**	**220**
**HBCDD**	**3.0**	**0.41**	**74**	**0.021**	**0.0031**	**0.098**	**0.00018**	**0.000039**	**0.00081**	**0.12**	**1.2**	**3.2**	**75**
**Σ_{20}BFRs**	**61**	**30**	**1,000**	**0.38**	**0.19**	**0.88**	**0.025**	**0.0033**	**0.095**	**7.9**	**50**	**69**	**1,100**

[a] sum of BDE-28, -47, -99, -100, -153, -154, and -183.

Table S9. Reference doses (RfD, ng/kg bw/day) for BFRs of interest,[8] and comparisons with the estimated daily intake (EDI) of BFRs for UK adults and toddlers

BFRs	RfD	EDI/RfD ratios for toddlers		EDI/RfD ratios for adults	
		Mean	High	Mean	High
BTBPE	243,000	< 0.001	< 0.001	< 0.001	< 0.001
EH-TBB	20,000	< 0.001	< 0.001	< 0.001	< 0.001
BEH-TEBP	20,000	< 0.001	0.0065	< 0.001	< 0.001
DBDPE	333,333	< 0.001	< 0.001	< 0.001	< 0.001
BDE-209	7,000	0.0059	0.12	< 0.001	0.0035
	(700) [a]	0.059	1.2	0.0029	0.035
Penta-BDE	2,000	< 0.001	< 0.001	< 0.001	0.0016
BDE-47	100	0.0010	0.0070	0.0030	0.023
BDE-99	100	0.0012	0.0083	0.0036	0.026
BDE-153	200	< 0.001	< 0.001	< 0.001	0.0047
∑HBCDDs	200,000	< 0.001	< 0.001	< 0.001	< 0.001

[a] For BDE-209, EPA has assigned an oral slope factor for carcinogenic risk of 700 $(ng/kg\ bw/day)^{-1}$.

Figures

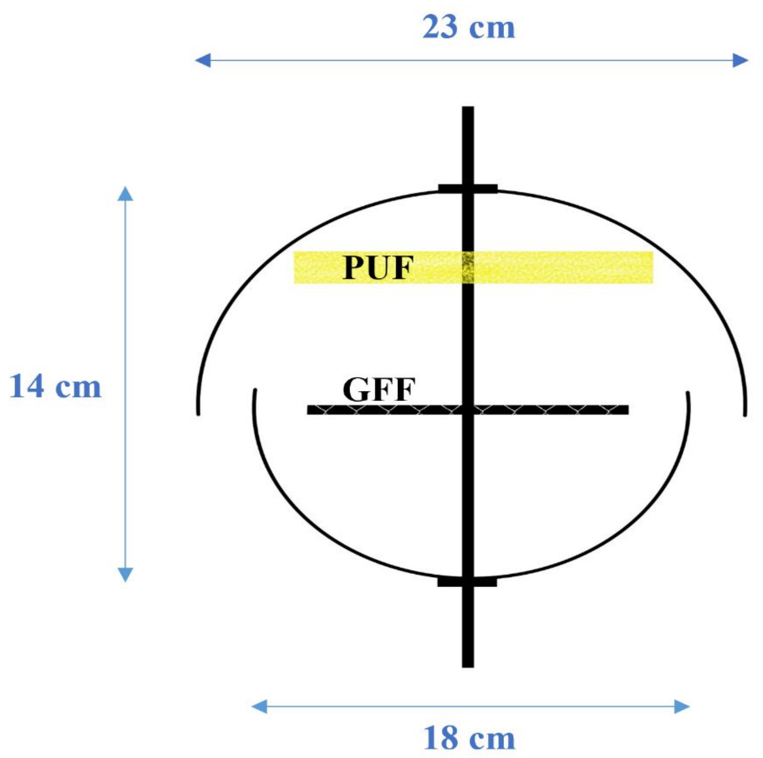

Fig. S1 Passive sampler configuration used for indoor and outdoor air sampling

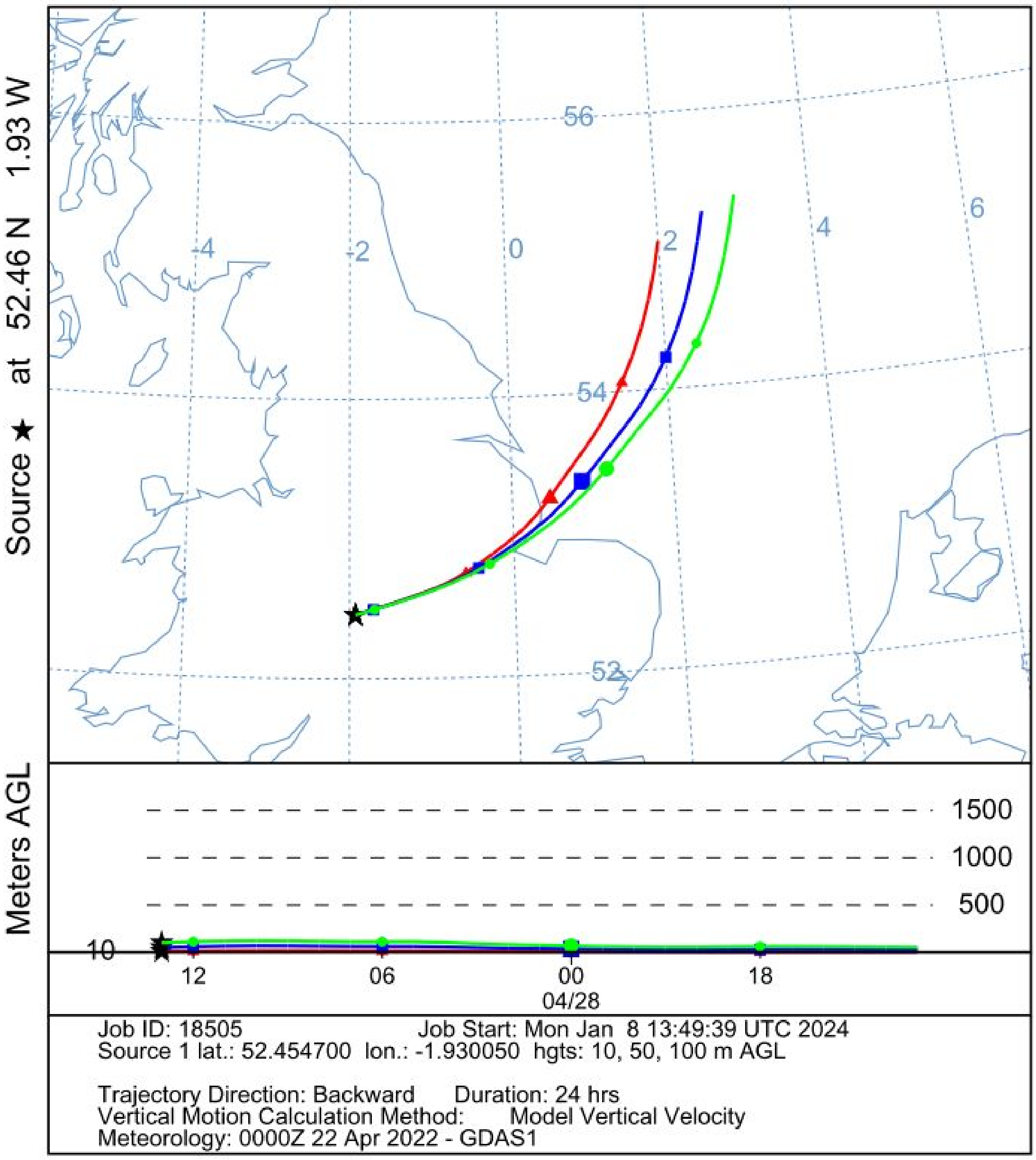

Fig. 2 Impact of southwest winds on atmospheric concentrations of BFRs (demonstrated by HYSPLIT model)

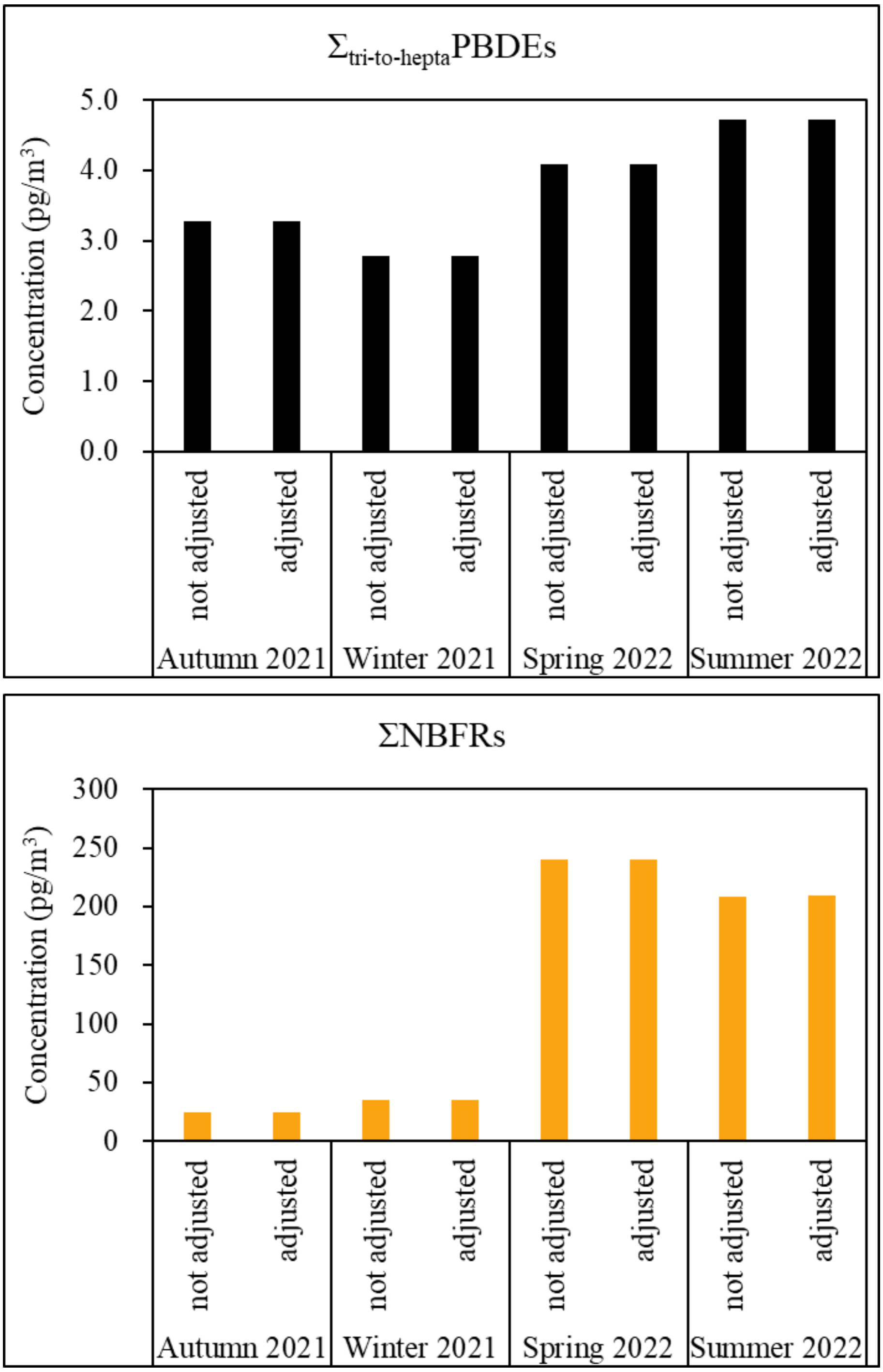

Fig. S3 Seasonal variations in atmospheric concentrations of BFRs: comparisons of BFR concentrations before vs after temperature-driven adjustments in air sampling rates of PAS

References

1. Tao, F.; Abdallah, M. A.; Harrad, S., Emerging and Legacy Flame Retardants in UK Indoor Air and Dust: Evidence for Replacement of PBDEs by Emerging Flame Retardants? *Environ Sci Technol* **2016,** *50*, (23), 13052-13061.

2. Abdallah, M. A.-E.; Harrad, S., Modification and calibration of a passive air sampler for monitoring vapor and particulate phase brominated flame retardants in indoor air: application to car interiors. *Environmental science & technology* **2010,** *44*, (8), 3059-3065.

3. Drage, D. S.; Newton, S.; de Wit, C. A.; Harrad, S., Concentrations of legacy and emerging flame retardants in air and soil on a transect in the UK West Midlands. *Chemosphere* **2016,** *148*, 195-203.

4. Harner, T., 2021 v10 Template for calculating PUF and SIP disk sample air volumes April28. **2021**.

5. Wilford, B. H.; Harner, T.; Zhu, J.; Shoeib, M.; Jones, K. C., Passive Sampling Survey of Polybrominated Diphenyl Ether Flame Retardants in Indoor and Outdoor Air in Ottawa, Canada: Implications for Sources and Exposure. *Environmental Science & Technology* **2004,** *38*, (20), 5312-5318.

6. NHS-Digital, Health Survey for England, 2019: Data tables https://digital.nhs.uk/data-and-information/publications/statistical/health-survey-for-england/2019/health-survey-for-england-2019-data-tables 20211017. **2019**. (date last accessed: March 19, 2022)

7. Ma, Y.; Stubbings, W. A.; Abdallah, M. A.-E.; Cline-Cole, R.; Harrad, S., Temporal trends in concentrations of brominated flame retardants in UK foodstuffs suggest active impacts of global phase-out of PBDEs and HBCDD. *Science of The Total Environment* **2023,** *863*.

8. Ma, Y.; Stubbings, W. A.; Cline-Cole, R.; Harrad, S., Human exposure to halogenated and organophosphate flame retardants through informal e-waste handling activities - A critical review. *Environmental Pollution* **2021,** *268*.
